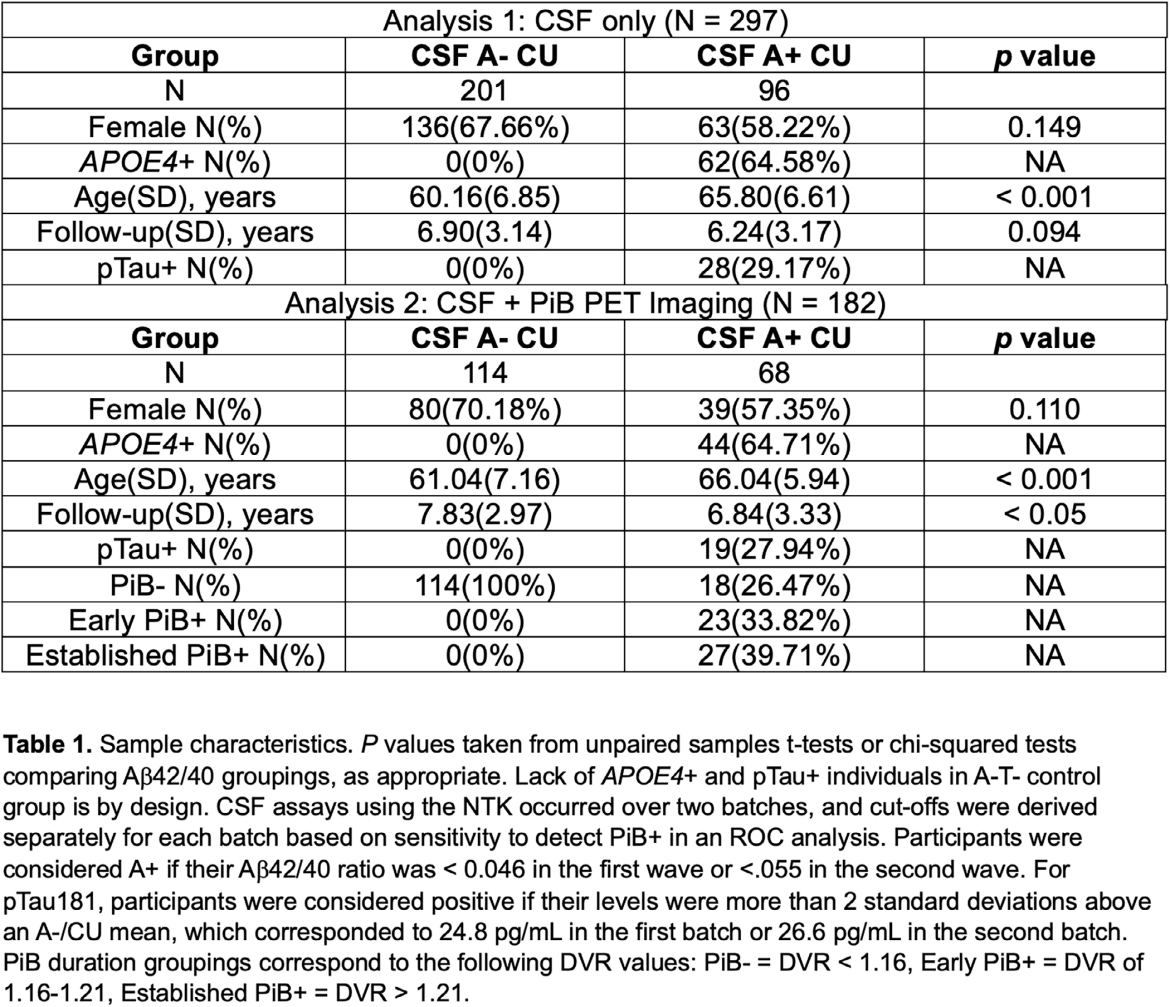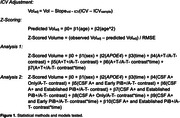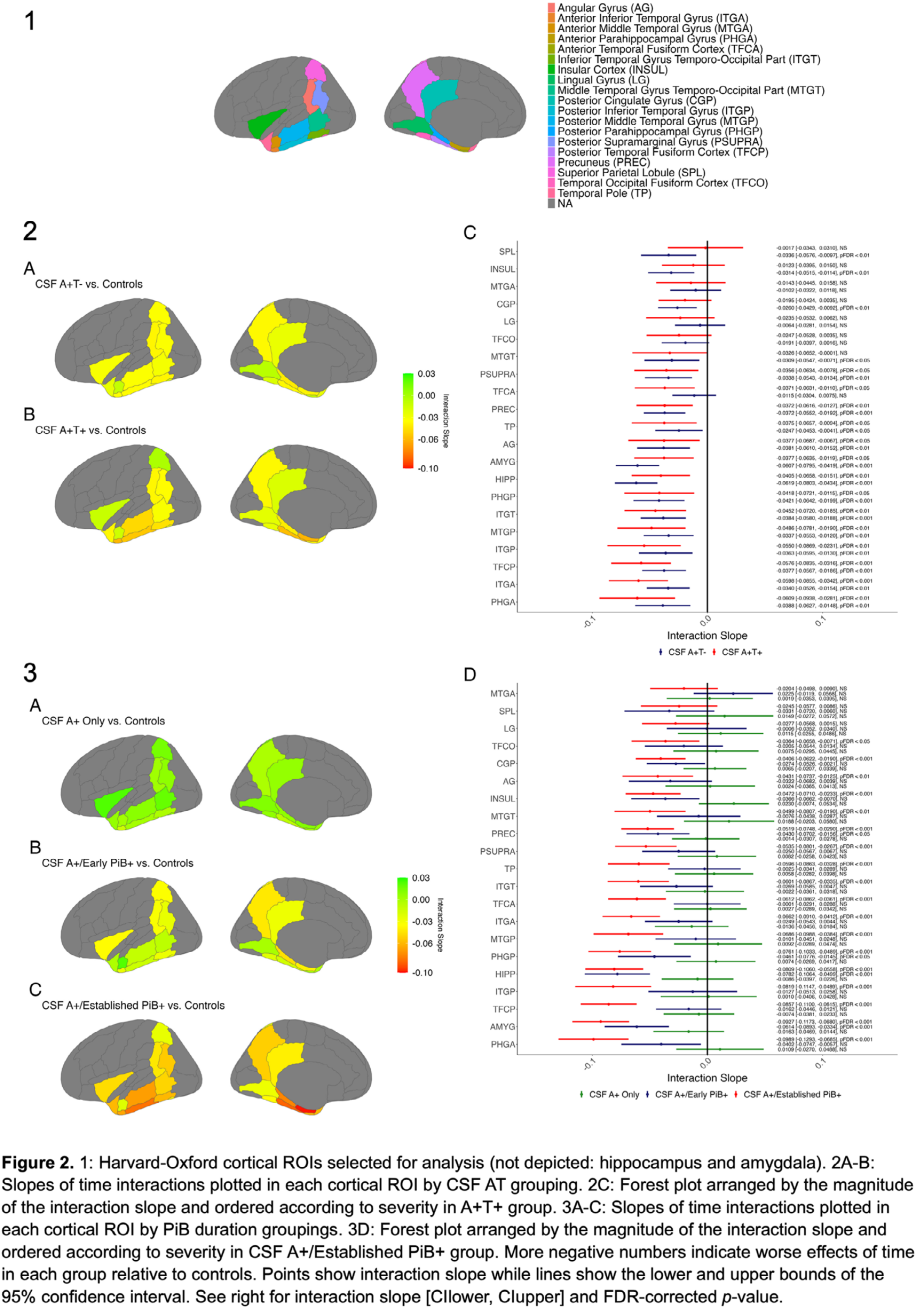# Longitudinal neurodegeneration in relation to amyloid pathology among cognitively unimpaired individuals

**DOI:** 10.1002/alz.092448

**Published:** 2025-01-09

**Authors:** Henry Gilreath Stephenson, Tobey J. Betthauser, Carol A. Van Hulle, Lianlian Du, Rebecca E. Langhough, Erin M. Jonaitis, Gwendlyn Kollmorgen, Clara Quijano‐Rubio, Nathaniel A. Chin, Ozioma C Okonkwo, Cynthia M. Carlsson, Sanjay Asthana, Bradley T. Christian, Sterling C. Johnson, Kaj Blennow, Henrik Zetterberg, Barbara B. Bendlin

**Affiliations:** ^1^ School of Medicine & Public Health, University of Wisconsin‐Madison, Madison, WI USA; ^2^ Neuroscience Training Program, University of Wisconsin‐Madison, Madison, WI USA; ^3^ Wisconsin Alzheimer's Disease Research Center, University of Wisconsin School of Medicine and Public Health, Madison, WI USA; ^4^ Wisconsin Alzheimer’s Institute, University of Wisconsin‐Madison School of Medicine and Public Health, Madison, WI USA; ^5^ Roche Diagnostics GmbH, Penzberg Germany; ^6^ Wisconsin Alzheimer's Disease Research Center, Madison, WI USA; ^7^ Wisconsin Alzheimer’s Institute, University of Wisconsin School of Medicine and Public Health, Madison, WI USA; ^8^ VA Geriatric Research, Education and Clinical Center (GRECC), William S. Middleton Memorial Veterans Hospital, Madison, WI USA; ^9^ Department of Medical Physics, University of Wisconsin‐Madison School of Medicine and Public Health, Madison, WI USA; ^10^ Department of Medical Physics, University of Wisconsin‐Madison, Madison, WI USA; ^11^ School of Medicine and Public Health, University of Wisconsin‐Madison, Madison, WI USA; ^12^ Department of Psychiatry and Neurochemistry, Institute of Neuroscience and Physiology, The Sahlgrenska Academy, University of Gothenburg, Mölndal Sweden; ^13^ Clinical Neurochemistry Laboratory, Sahlgrenska University Hospital, Mölndal Sweden; ^14^ Department of Neurodegenerative Disease, UCL Institute of Neurology, London United Kingdom; ^15^ UK Dementia Research Institute, University College London, London United Kingdom; ^16^ Hong Kong Center for Neurodegenerative Diseases, Clear Water Bay Hong Kong

## Abstract

**Background:**

The timing of neurodegeneration in relation to the onset of Alzheimer’s disease pathology is not fully known. This study examined the association of longitudinal atrophy derived from T1‐weighted MRI with 1) cerebrospinal fluid (CSF) amyloid‐tau (AT) groupings and 2) Pittsburgh compound B (PiB) PET‐derived estimates of amyloid duration among cognitively unimpaired (CU) individuals.

**Method:**

CU participants in the Wisconsin Registry for Alzheimer’s Prevention and Wisconsin Alzheimer’s Disease Research Center (N = 297) underwent longitudinal MRI, APOE genotyping, and lumbar puncture to determine CSF Aβ42/40 (A) and pTau181 (T) concentration at baseline using in‐house cutoffs. CSF measurements were performed using the NeuroToolKit, a panel of robust prototype biomarker assays (Roche Diagnostics International Ltd). Image segmentation was performed using a longitudinal pipeline in SPM12. Grey matter volumes from AD‐associated regions (adjusted for intracranial volume) were z‐scored according to age‐associated predictions from a robust longitudinal A‐T‐/APOE4‐/CU control group. Differences in longitudinal atrophy relative to controls were tested in linear mixed‐effects models using contrasts between A+T‐ and A+T+ groups and A‐T‐ controls and interactions with time (years from baseline). In the PiB PET subset, sampled iterative local approximation was used to estimate time‐to‐positivity at baseline relative to a PiB DVR threshold of 1.16. CSF A+ individuals were grouped as CSF A+ only, early PiB+ (PiB+ <= 5 years), and established PiB+ (PiB+ > 5 years). Differences in longitudinal atrophy were tested with A+ group/A‐T‐ contrasts and interactions with time. All analyses were FDR‐corrected. See Table 1 and Figure 1 for participant characteristics and model details.

**Result:**

Based on CSF assays, both A+ groups showed longitudinal atrophy relative to controls in a majority of ROIs, suggesting that A+, regardless of pTau+, is associated with neurodegeneration. Further analysis incorporating PET showed that CSF A+ in the absence of PiB+ was not associated with atrophy, but early PiB+ was associated with atrophy in medial temporal ROIs and precuneus. The largest and most widespread atrophy was observed in the established PiB+ group (Figure 2).

**Conclusion:**

Neurodegeneration appears to begin soon after the onset of PET‐measurable amyloid‐positivity. Clinical intervention at the earliest signs of amyloid deposition may be needed to delay neurodegeneration.